# Visualizing Reentry Vulnerable Targets During Scar-Related VT Ablation: A Novel Functional Substrate Mapping Approach Integrating Conduction and Repolarization Metrics

**DOI:** 10.1161/CIRCEP.124.012915

**Published:** 2024-06-28

**Authors:** Johanna B. Tonko, Anthony Chow, Cristina Lozano, Javier Moreno, Pier D. Lambiase

**Affiliations:** 1 Institute for Cardiovascular Science, University College London, United Kingdom (J.B.T., P.D.L.).; 2 Barts Heart Centre, St Bartholomew s Hospital, London, United Kingdom (J.B.T., A.C., P.D.L.).; 3 Department of Cardiology, Hospital Universitario Ramón Y Cajal, Madrid, Spain (C.L., J.M.).

**Keywords:** electrophysiological phenomena, retrospective studies, sudden cardiac death, therapy, ventricular tachycardia

Combined visualization of conduction and repolarization metrics during substrate mapping in the form of a reentry vulnerability index (RVI) may enable more precise identification of critical ablation targets. The RVI is based on previous studies demonstrating the likelihood of excitation wavefront-waveback interactions between 2 points across a line of conduction block following a premature stimulus.^1^ Retrospective RVI computation of postoperatively analyzed substrate maps showed promising results for locating ventricular tachycardia (VT) exit sites.^2^ However, RVI mapping has not been used in clinical cases due to the requirement of extensive offline postprocessing for its derivation.

Here, we present a workflow to generate high-density repolarization maps and integrate conduction and repolarization metrics to visually estimate reentry vulnerable sites of adjacent long activation time (AT) and short repolarization time in the mapping system. We retrospectively applied this mapping strategy to high-density mapping datasets of previous clinical VT ablation procedures to relate visual RVI zones to functionally critical cites of the VT circuit. The study was approved by the institutional review boards, and patient consent was obtained. Data to support the findings are available upon reasonable request.

First, deceleration zones, areas of late activation, and lines of block were tagged on omnipolar local activation timing substrate maps. Then, repolarization maps were generated based on the corresponding electrograms (EGMs) collected during the initial substrate map using the TurboMap^TM^ feature (Ensite^TM^ X, Abbott). The window of interest was set over the T wave, and the detection criteria were set to the maximal upslope (+dV/dt) thus annotating local repolarization time according to the Wyatt method on the unipolar T wave (Figure A). The omnipolar conduction (local AT) and unipolar repolarization maps (local repolarization time [RT]) were displayed side-by-side. A visual RVI zone was identified based on color scales if an area of early, that is, short repolarization time (defined as within 100 ms of the latest local activation timing point), overlapped and/or was directly adjacent to a tagged area of late activation. In visually identified RVI areas, the temporal and spatial relationship of EGMs with the earliest RT to EGMs with the latest AT separated by a maximum of 20 mm were measured and RVI calculated (=earliest RT−latest AT). The lower, respectively more negative, the RVI is, the higher the reentry vulnerability. Thereafter, distances from visual RVI zones to functionally critical zones were measured (Figure B).

**Figure.**
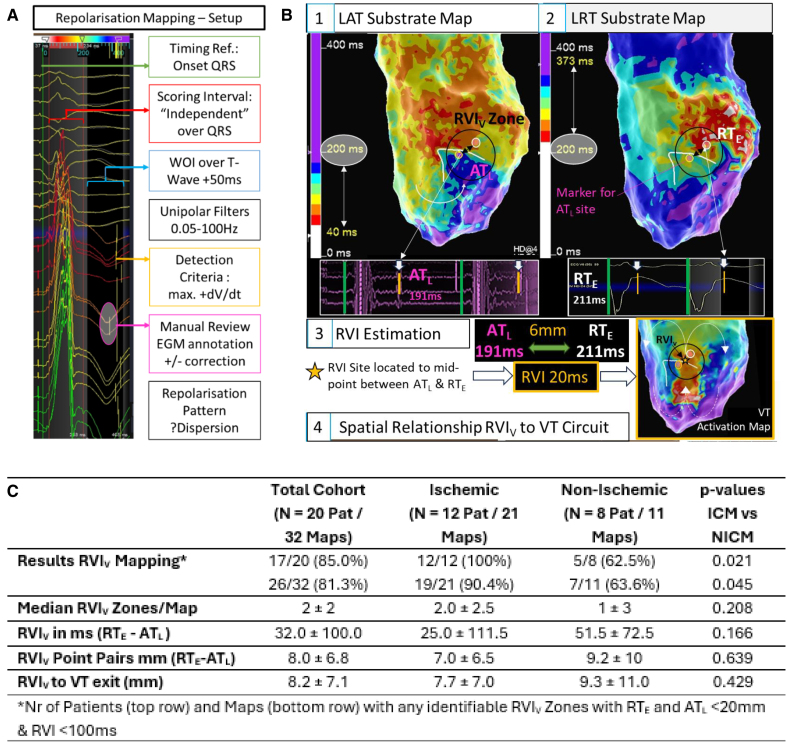
**Overview repolarization and reentry vulnerability mapping. A**, Step-by-step setup of a repolarization map using unipolar EGMs. **B**, Visual RVI estimation workflow (1) LAT substrate map using last deflection for LAT detection; (2) LRT via TurboMap^TM^ of collected data points from activation map annotating RT to the steepest upslope of the T wave (max +dV/dt); (3) latest activation on color scale of LAT map (white circle in panel 1) is used to define threshold for early repolarization on color scale of LRT (white circle in panel 2) and facilitate identification of area of late activation (blue/purple) adjacent to areas of early repolarization (white/red). Visual RVI zones can be rapidly identified if markers of blue/purple late activation overlap and/or are adjacent to white/red early repolarization sites. To quantify reentry vulnerability, a numeric RVI can be estimated by subtracting the latest AT duration from the earliest RT duration and measuring the distance between these EGM sites. (4) For the purpose of this study, the spatial relationship between RVI_V_ sites and the VT circuit was evaluated. **C**, Overview of the results of RVI mapping applied to a scar-related VT cohort. AT indicates activation time; AT_L_, latest activation time; iLAM, isochronal late activation mapping; LAT, local activation time; LOB, line of block; LRT, local repolarization time; RT, repolarization time; RT_E_, earliest repolarization time; RVI, reentry vulnerability index; RVI_V_, visual reentry vulnerability _index_; VT, ventricular tachycardia ; and WOI, window of interest.

Twenty scar-related reentry VT cases (90% male, 63.8±11.6 years, 60% ischemic, LVEF 36±16%) were reviewed. In 17 of 20 (85%) patients, at least 1 reentry vulnerable zone was identified on the substrate map. Three cases without identifiable visual RVI despite endo-epicardial mapping were of nonischemic etiology with presumed midmyocardial substrate. Manually calculated RVIs were 32.0±100 ms (most vulnerable RVI, −227 ms) with a distance of 8.0±6.8 mm between the earliest RT and latest AT EGMs. The shortest distance of any visual RVI to functionally critical VT sites defined as sites of VT termination during ablation or entrainment (65%) or pace mapping (35%) was 8.2±7.1 mm.

In 18 of 20 patients (90%) and 26 of 32 maps (81.3%), a deceleration zone was evident (on average, 1.7±0.9 per map). In 25 of 32 maps (78%), at least 1 localized line of block was identified, and visual RVI sites were located on average 3.0±6.0 mm from a LOB. VT exit sites were mapped 5.1±3.9 mm to a LOB.

The presented pilot data indicate that with our workflow, it is feasible to create high-resolution repolarization maps and identify reentry vulnerable sites without the need for VT induction or more complex computational data processing. The assessment of repolarization abnormalities in routine clinical procedures would be an important step forward in physiologically based mapping for scar-related VTs. Future work needs to focus on improving the accuracy of repolarization mapping and addressing current limitations (ie, susceptibility to noise distortion of unipolar EGMs, lack of mapping features/filtering dedicated to repolarization dynamics). Assessing the specificity of this novel approach is challenging as it would require relating all inducible VTs to each RVI site compared with a traditional substrate feature (eg, ILAM and lines of block), which is often not feasible in a clinical study, and also not all VTs may be inducible during the procedure. A prospective study comparing ILAM mapping versus RVI could be undertaken to assess the number of lesions required for noninducibility and assess clinical outcomes with either strategy. Larger studies could also refine the temporal and spatial thresholds of the latest AT–earliest RT EGM pairs used for RVI estimation to fully define the degree of vulnerability of RVI that warrants targeted ablation.

In conclusion, visual reentry vulnerability estimation including novel repolarization maps is feasible using clinical mapping tools. This may support localization of functionally critical zones to target ablation without the requirement for VT induction and potentially reduce the amount of ablation required to eliminate VT circuits.

## ARTICLE INFORMATION

### Sources of Funding

None.

### Disclosures

Dr Lambiase is supported by University College London Hospital Biomedicine National Institute for Health and Care Research and Barts Biomedical Research Centre. Dr Tonko acknowledges Fellowship support from Bart s Charity London, United Kingdom. Dr Moreno receives speaker fees from Abbott, Biosense Webster, and Boston Scientific. The other authors report no conflicts.
